# Early Shedding of Sepals Promotes Cross-Pollination of *Actaea erythrocarpa* (Ranunculaceae)

**DOI:** 10.3390/biology15060468

**Published:** 2026-03-14

**Authors:** Jiudong Zhang, Weijun Xu, Deng Yang, Xiaoxiao Liu, Xiaohui Zhang, Rui Guo, Jing Xu, Ziwei Li, Jie Sui, Lin Wang, Tianpeng Gao

**Affiliations:** 1 School of Biological and Environmental Engineering, Xi’an University, Xi’an 710065, China; zhangjiudong029@163.com (J.Z.); hangxiaosu618@163.com (W.X.); xiaoxiaoliulg@163.com (X.L.); jing.xu@xawl.edu.cn (J.X.); liziwei0427@163.com (Z.L.); 2 Shaanxi Provincial Aerospace Breeding Engineering Technology Research Center, Xi’an 710065, China; htyzguorui@163.com; 3 College of Life Sciences, Shaanxi Normal University, Xi’an 710119, China; xhzhang@snnu.edu.cn (X.Z.); jidelikeyiyi@163.com (J.S.); wangmumu234@163.com (L.W.); 4 College of Ecology, Lanzhou University, Lanzhou 730030, China; ydeng2024@lzu.edu.cn; 5 Engineering Center for Pollution Control and Ecological Restoration in Mining of Gansu Province, Lanzhou City University, Lanzhou 730070, China

**Keywords:** breeding system, pollination biology, fly insects, floral characteristics

## Abstract

The diversity of floral traits in angiosperms has evolved over time as an adaptive response to reproductive demands. Investigating floral characteristics and pollination systems helps elucidate the evolutionary drivers behind morphological variation in flowers. In this study, we examined *A. erythrocarpa* to systematically document its floral morphology, stigma receptivity, pollen–ovule ratio, breeding system, and pollinator behavior. Results showed that the sepals abscised completely at the early flowering stage, while stigmas became receptive even during the bud phase. The pollen–ovule ratio was 1773.58 ± 689.75, indicating a facultatively xenogamous breeding system. Bagging experiments further confirmed that the species is self-compatible but does not exhibit apomixis or wind pollination. Field observations identified *Agromyzidae* sp. and *E. balteatus* as the primary pollinators, which visited flowers at high frequency after sepal abscission but before anther dehiscence. Compared with its congener *A. asiatica*, which exhibits delayed sepal abscission and relies mainly on selfing, *A. erythrocarpa* demonstrates distinct floral morphological adaptations linked to its specialized pollination strategy. These two species thus represent divergent reproductive adaptation patterns within the genus. Therefore, the visiting behavior of fly insects and the early shedding of sepals promotes cross-pollination of *A. erythrocarpa*.

## Simple Summary

*Agromyzidae* sp. and *Episyrphus balteatus* De Geer are the main pollinators, and they have a high frequency of visiting flowers after the sepals have fallen off and the anthers have not yet opened. A comparison of sepal morphology between *Actaea asiatica* Hara, which undergoes self-pollination, and *Actaea erythrocarpa* Fisch., which undergoes cross-pollination, suggests that the variation in sepal characteristics is closely related to the breeding system. The sepals of *Actaea erythrocarpa* fall off when the flowers just open and the stigma is receptive at the bud stage, which belongs to the breeding system of facultative crossbreeding. Therefore, the visiting behavior of fly insects and the early shedding of sepals promotes cross-pollination in *A. erythrocarpa*.

## 1. Introduction

The Ranunculaceae family exhibits remarkable diversity and complexity in floral morphology and pollination mechanisms, and cross-pollination has evolved from generalization to specialization. Many studies have focused on the adaptability of petals and insects, such as the co-evolutionary relationship between floral spurs in *Aquilegia* and the mouthparts of their pollinators [1,2,3], the specialized adaptation of zygomorphic flowers in *Aconitum* to bumblebee pollination [4], and the adaptability of *Actaea purpurea* between hornet species and purple flower organ [5], and the deception of flies by the false nectaries of *Eranthis stellataon* Maxim. as well as the honey-guiding effect on bees [6]. In contrast, floral characteristics in the genus *Actaea* within the same family are generally more conservative. The flowers are mostly radially symmetrical, with limited variation in corolla color, petals, stigmas, and stamens. This genus is widely distributed across the Northern Hemisphere [7]. In China, only two species are present: the widely distributed *A. asiatica* and the narrowly distributed *A. erythrocarpa*, which is restricted to regions such as Shanxi, Inner Mongolia, and Jilin [8]. Existing studies have revealed variations in the breeding systems among different *Actaea* species across regions. For example, populations of *A. erythrocarpa* in North America and Russia exhibit a facultatively outcrossing breeding system, while parthenogenesis has been observed in Finnish populations of *A. rubra* [9]. Additionally, *A. pachypoda* and *A. spicata* are typically insect-pollinated outcrossing species. Regarding *A. asiatica* in China, Zhang et al. found that under conditions of pollinator scarcity, it can achieve autonomous selfing through delayed sepal abscission [10].

The phylogenetic tree of Compton et al. (1998) treated *A. rubra* and *A. erythrocarpa* as the same species [7]. However, the specific reproductive strategy of *A. erythrocarpa* distributed in China remains unclear. How do its key floral traits, particularly sepal characteristics and breeding system, differ from those of the predominantly selfing *A. asiatica*? To address these questions, we make assumptions that ‘early sepal abscission promotes cross-pollination’, which aims to conduct a systematic investigation into the pollination biology and breeding system of *A. erythrocarpa*. By comparing its sepal traits with those of its close relative *A. asiatica*, we seek to clarify its cross-pollination mode and reproductive adaptation mechanisms. This will promote our understanding, within the context of changing pollination environments, of the ecological and evolutionary significance of subtle floral morphological variations for plant reproductive success.

## 2. Materials and Methods

### 2.1. Experimental Materials and Study Sites

This study selected *A. erythrocarpa* and *A. asiatica* as research subjects, with the experiments conducted at two separate locations. The study site for *A. erythrocarpa* is located in the Changbai Mountains, Jilin Province (42°03′32.23″ N, 128°03′18.50″ E; elevation 1827 m). During the period from June 10 to 30 in each year from 2023 to 2025, two 20 m × 20 m plots spaced more than 500 m apart were established in this area. Systematic investigations were carried out on floral morphology, flowering phenology, breeding system, and pollination biology. Experimental materials of *A. asiatica* were collected from Taibai Mountain, Mei County, Shaanxi Province (34°24′51.48″ N, 107°41′49.74″ E; elevation 1339 m).

### 2.2. Floral Traits and Flowering Dynamics

To observe the sequence of flower opening, twenty individuals of *A. erythrocarpa* were randomly selected, ten individuals with unopened inflorescences were selected, and thirty fully opened flowers were chosen to measure the length of the inflorescence axis and the number of individual flowers on each axis. A vernier caliper was used to measure the diameter of the open flower, the lengths of the stamens and pistils, and the length and width of the petals and sepals. The floral organs were also dissected to count their numbers. For the observation of flowering dynamics, five plants were chosen, and five flower buds of similar developmental stages were marked on each plant. The opening status and the number of opened individual flowers on the main inflorescence axis were recorded daily at 08:00, 12:00, and 16:00. On the day of anthesis, observations were conducted hourly to record the sequence and dynamic changes in floral organ opening. After the sepals had fully expanded (approximately 24 h after anthesis), observations were made daily at 08:00, 10:00, 12:00, 14:00, and 16:00. Data recorded included floral shape, the duration of opening for each organ (sepals, petals, stamens, and pistils), the maturation times of the stamens and pistils, and the sequence of senescence of the floral organs.

### 2.3. Breeding System Assessment

To assess the breeding system of *A. erythrocarpa*, ten individuals with unopened inflorescences were randomly selected prior to another dehiscence, all anthers from a single flower were collected, placed in a 10 mL centrifuge tube, and crushed. Then, 5 mL of purified water was added to disperse the pollen grains. The pollen number was counted using a hemocytometer under a optical E200 microscope (Nikon, Tokyo, Japan), while the ovule number per flower was tallied under a stereomicroscope (Olympus, Tokyo, Japan). Stigma receptivity was determined using the benzidine-hydrogen peroxide method [11], and pollen viability, along with its duration, was tested via the MTT method [12]. The breeding system was preliminarily evaluated based on the pollen–ovule ratio (P/O) proposed by Cruden and the outcrossing index described by Dafni [11,13].

A hand-pollination experiment was conducted to examine mating patterns. Twenty inflorescences were selected, with three flower buds marked on each, resulting in a total of 60 buds. At the bud stage or just before anthesis, the following six treatments were applied: (1) no treatment (control); (2) removal of stamens to test for apomixis; (3) emasculation followed by bagging with an 8-mesh net to test for wind pollination; (4) self-pollination with pollen from the same flower to test for self-compatibility; (5) emasculation before anthesis followed by pollination with pollen from a different flower on the same plant; and (6) emasculation before anthesis followed by pollination with pollen from a different individual. The wind pollination treatment uses 8-mesh nylon bags with a mesh size of 40 * 60 mm, all other groups were immediately enclosed in 300-mesh nylon bags after pollination. During the period of stigma receptivity, flowers in groups (4), (5), and (6) were pollinated daily between 10:00 and 12:00 for three consecutive days. After fruit maturation, seed set was recorded for all treatment groups.

Seed-set rate = number of mature seeds/average ovule number

### 2.4. Pollinator Observation

Observations of flower-visiting insects were conducted following the method described by Dafni [11,13]. In each study plot, three populations were selected, and within each population, 3–5 inflorescences of *A. erythrocarpa* were chosen for observation. Observation periods were set during the daytime (09:00–17:00), with additional monitoring at night (20:00–00:00) to detect any nocturnal flower visitors. Data recorded included: the visitation frequency of each insect type per unit time, their foraging paths on individual flowers or inflorescences, and the time spent crawling and feeding on flowers.

During insect visits, six individuals of each insect type were collected. Three of these were preserved in 75% ethanol for taxonomic identification, while the remaining three were air-dried for pollen load analysis. The dried specimens were examined under a stereomicroscope to observe pollen grains adhering to body parts such as the thorax, abdomen, and legs. Subsequently, the insects were sputter-coated with gold and photographed using a Hitachi S-570 scanning electron microscope (Hitachi, Tokyo, Japan) to quantify the pollen loads on different body regions of each insect type.

### 2.5. Data Analysis

All data were analyzed using PRISM10.1.2 software. To test was employed to examine differences in seed-set rates among different pollination treatments, variations in pollinator visitation frequency across flowering stages, and the effects of floral morphological traits and floral rewards on insect visitation frequency by one-way ANOVA.

## 3. Results

### 3.1. Morphological Traits and Flowering Dynamics

**Figure 1:**
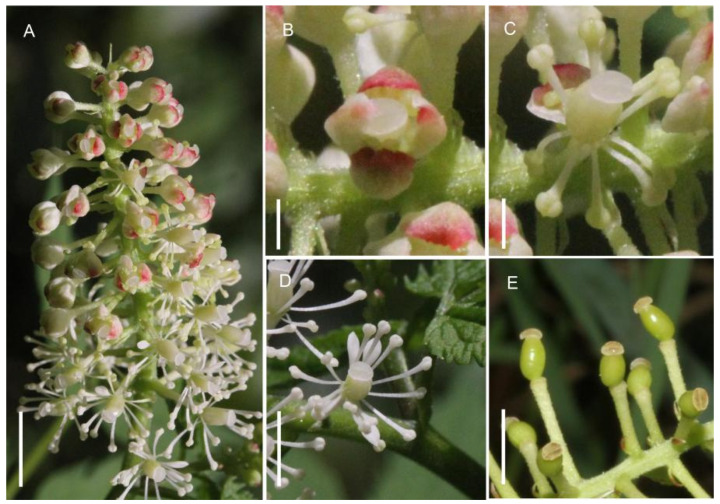
Inflorescence characteristics and main pollinating insects of *A. erythrocarpa*. (**A**) Inflorescence, shows that the single flower gradually opens from the base to the top. (**B**–**E**) Single flower opening process. (**B**) Flower bud tip cracked. (**C**) The sepals all fall off, exposing other floral organs. (**D**) Stamens and petals spread out. (**E**) Except for the pistil, all other floral organs fall off. Bar = 6 mm.

The main inflorescence axis of *A. erythrocarpa* measured 31–82 mm in length and bore 26–68 densely arranged flowers. The diameter of a single open flower ranged from 8.06 to 12.21 mm. Each flower possessed four sepals, with pinkish apices, while the remaining floral organs were white (Figure 1A–D). The sepals were 2.03–3.25 mm long and 0.98–2.34 mm wide. Within the same inflorescence, individual flowers had 1–4 petals, measuring 1.16–2.63 mm in length and 0.6–1.22 mm in width. Stamens numbered 10–13 and were 3.54–7.13 mm long; the single pistil measured 3.05–3.48 mm, approximately two-thirds the length of the stamens. Within a flower, the stamens extended outward, maintaining spatial separation from the stigma, thus creating spatial isolation between the male and female organs.

The flowering period of the population began in mid-June and ended in early July, lasting 20–25 days. Flowers on the same inflorescence opened sequentially from the base toward the apex (Figure 1A), with individual flower longevity of 5–6 days. At anthesis, one sepal often abscised first due to elongation of the petals or stamens, while the remaining sepals abscised completely within one day after flower opening (Figure 1B,C). Within one day after sepal abscission, the stamens began to elongate erect (Figure 1D). Anthers dehisced from the inner to outer whorls, and pollen release lasted 3–4 days. After pollen dispersal, all floral organs except the stigma senesced and fell (Figure 1E).

*A. erythrocarpa* exhibits dichogamy with protogyny. The stigma became receptive even at the bud stage, reached peak receptivity 2–3 days after anthesis, and then gradually declined, losing receptivity completely only after all stamens had senesced. Pollen viability was highest immediately after anther dehiscence, dropped below 50% within 48–56 h after dispersal, and was almost completely lost 84–96 h after dispersal.

**Figure 2:**
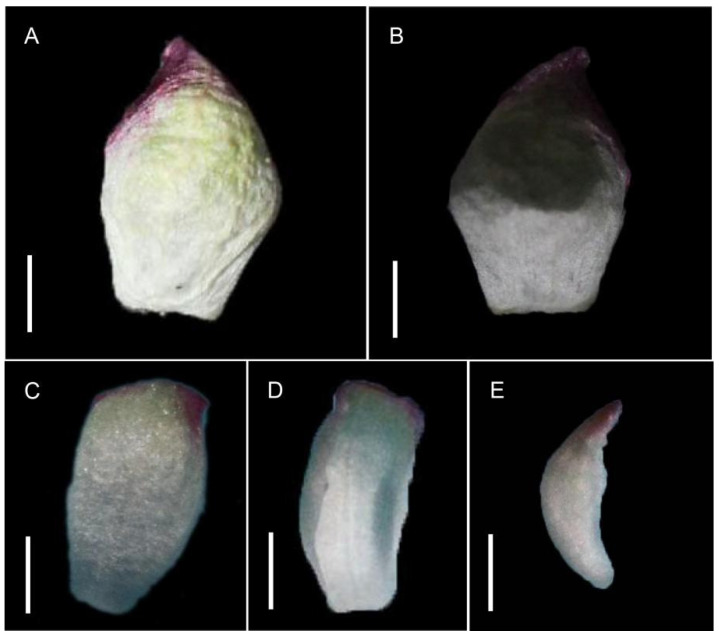
The sepals of *A. erythrocarpa* and *A. asiatica.* (**A**,**B**) Sepals of *A. asiatica*, length-to-width ratio 1:1, apex acuminate and inwardly curved. (**C**–**E**) Sepals of *A. erythrocarpa*. (**C**,**D**) Sepals truncated at both ends. (**E**) Sepals fold inward on both sides, acuminate at both ends. Bar = 1 mm.

Compared with its congener *A. asiatica*, sepal morphology differed markedly. In *A. asiatica*, all four sepals were uniform in shape, with acuminate apices, a length-to-width ratio of approximately 1:1, and inwardly curved tips (Figure 2A,B). In *A. erythrocarpa*, two sepal morphologies were observed: one type was longer than wide, with nearly parallel sides that curved slightly inward, enclosing the inner floral organs (Figure 2C,D); the other type was asymmetrical, folded inward laterally, and tapered at both ends (Figure 2E).

### 3.2. Assessment of the Breeding System

The flower diameter of *A. erythrocarpa* was 10.03 ± 1.44 mm. The species exhibits protogyny and spatial separation between pistils and stamens. Its outcrossing index was 4, indicating a predominantly outcrossing breeding system with partial self-compatibility and a dependence on pollinators (Table 1). The pollen–ovule ratio (P/O) was 1773.58 ± 689.75, further supporting a facultatively outcrossing breeding system (Table 1). Controlled hand-pollination experiments showed that self-pollination produced seeds with a seed-set rate of 83.78%, while geitonogamous pollination yielded a seed-set rate of 88.54%. No evidence of wind pollination or apomixis was detected, and there was no significant difference in seed-set rates between self and cross-pollination treatments (Table 2). In conclusion, based on the outcrossing index, pollen–ovule ratio, and the observation of abundant insect visitation in the field, *A. erythrocarpa* is confirmed to possess a predominantly outcrossing breeding system.

### 3.3. Pollinators

**Figure 3:**
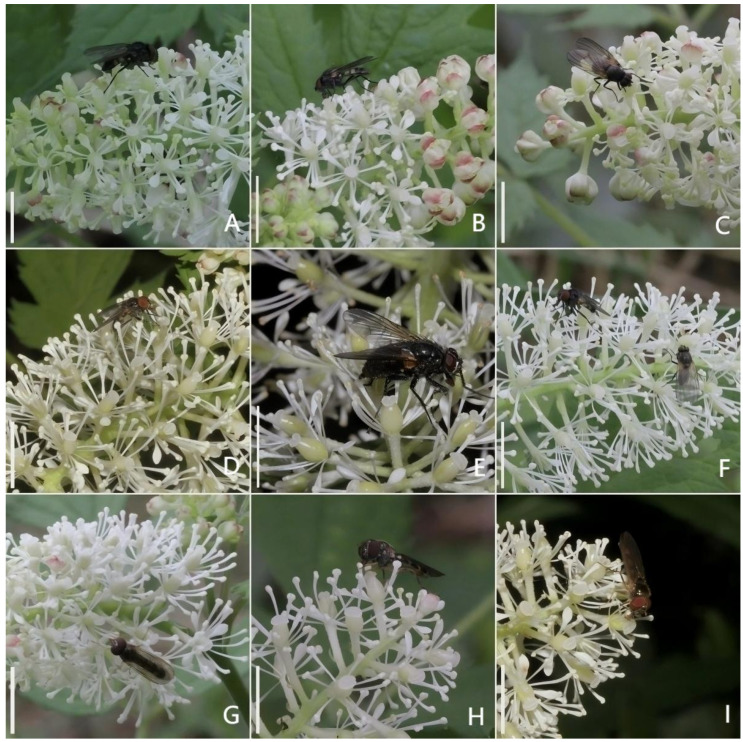
The flower visiting behavior of two main insects of *A. erythrocarpa.* (**A**–**C**) *Agromyzidae* sp. visits uncracked anthers. (**D**–**F**) *Agromyzidae* sp. visits cracked anthers. (**G**) *E. balteatus* visits uncracked anthers. (**H**,**I**) *E. balteatus* visits cracked anthers. Bar = 10 mm.

Floral visitors of *A. erythrocarpa* included insects from the families Agromyzidae, Sarcophagidae, *Episyrphus balteatus* (Syrphidae), Histeridae, and Culicidae. No nocturnal visitors were observed. Among these, dipterans of the family Agromyzidae (Figure 3A–F) were the primary pollinators. These insects possess sponging mouthparts and feed on pollen. They began visiting flowers even at the early opening stage when stamens had not yet dehisced (Figure 3A–C). Their typical foraging behavior involved crawling along the inflorescence to feed on pollen, often using stigmas and filaments as support points while moving within or between individual flowers. They usually revisited the same inflorescence 2–3 times before moving to another. During visits, their forelegs frequently clasped the filaments. Agromyzid flies showed high visitation frequency, ranging from 55 to 85 visits per inflorescence per day, with an average of 8.96 visits per flower per day. Another important pollinator was the hoverfly *E. balteatus* (Figure 3G–I), which also fed on pollen. While crawling and feeding, its body frequently contacted both stigmas and pollen. Its visitation frequency was 10–25 visits per inflorescence per day, averaging 2.16 visits per flower per day.

**Figure 4:**
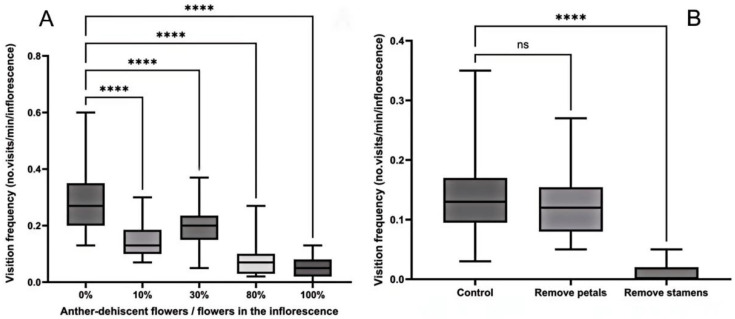
Relationship between flowering dynamics and visiting frequency of pollinators. (**A**) The relationship between the proportion of flowers of anther dehiscence in the inflorescence and the visit frequency of pollinators. (**B**) Effect of removal of floral organs on the frequency of pollinators. **** indicates significant differences, ns indicates no significant difference.

Further analysis revealed that both insect groups exhibited high visitation frequencies even before anther dehiscence began on the inflorescence. The frequency of fly visits to flowers with unopened anthers differs significantly from visits to flowers with opened anthers. (Figure 4A). Additionally, petal removal did not significantly affect insect visitation frequency, while stamen removal completely eliminated insect visits (Figure 4B).

**Figure 5:**
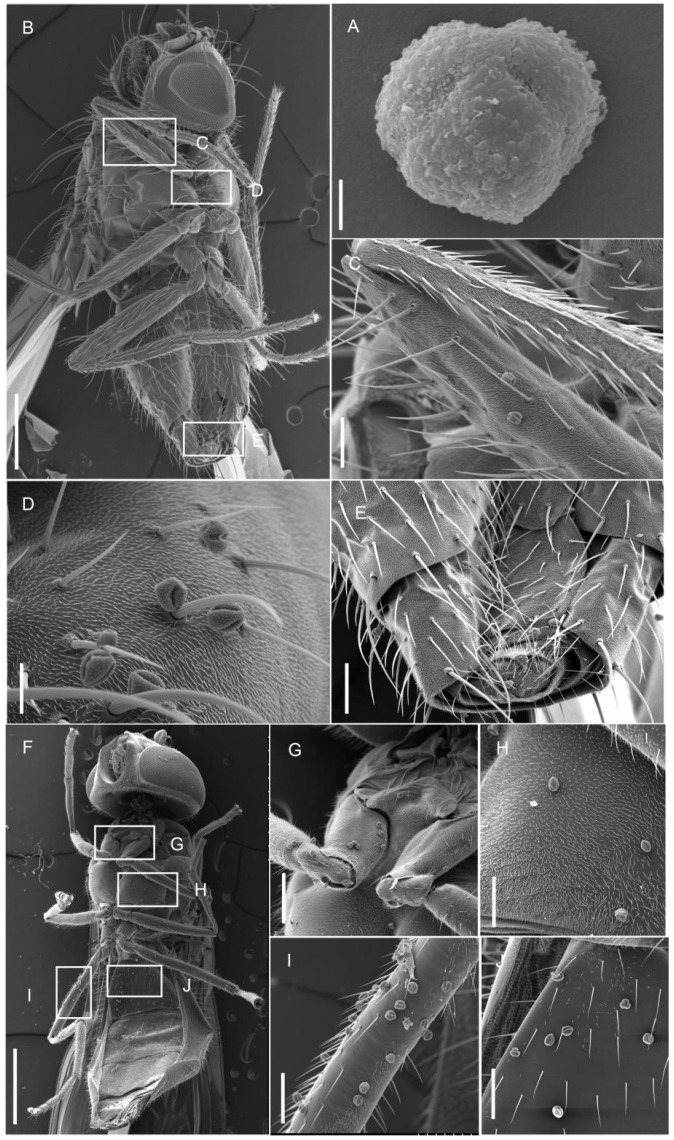
Pollinator carry pollen of *A. erythrocarpa*. (**A**) Pollen of *A. erythrocarpa*. (**B**–**E**) Carry pollen of *Agromyzidae* sp. (**B**) The SEM of *Agromyzidae* sp. (**C**) carry pollen of legs. (**D**) carry pollen of chest. (**E**) Carry pollen of abdomen. (**F**–**J**) Carry pollen of *E. balteatus*. (**F**) The SEM of *E. balteatus*. (**G**) Carry pollen of forefoot. (**H**) Carry pollen of chest. (**I**) Carry pollen of the third pair of feet. (**J**) Carry pollen of abdomen. (**A**) Bar = 5 um; (**B,F**) = 1 mm; (**C**–**E**,**G**–**J**) Bar = 50 um.

The pollen of *A. erythrocarpa* is tricolpate (Figure 5A). Scanning electron microscopy showed that agromyzid flies carried pollen grains on their legs, thorax, and abdomen (Figure 5B–E), with a load of 5–10 grains. *E. balteatus* carried pollen on its forelegs, midlegs, hind legs, thorax, and abdomen (Figure 5F–J), with loads of 5–20 grains on different body parts. Although its visitation frequency was lower than that of agromyzid flies, *E. balteatus* carried more pollen grains per visit. Therefore, both insects are effective pollinators of *A. erythrocarpa*. Histerid beetles were also observed visiting flowers, but no pollen was detected on them under scanning electron microscopy.

## 4. Discussion

Within the genus *Actaea* Linn. of the Ranunculaceae, *A. pachypoda*, *A. spicata*, *A. asiatica*, and *A. erythrocarpa* (*A. rubra*) are closely related [7,15]. Regarding pollination, these closely related species share weevils and small flies as their main pollinators [9,14], though the specific insect groups involved may differ. Although beetles were also observed visiting flowers, they carried no pollen and thus were ineffective as pollinators. In contrast, *A. spicata*, *A. rubra*, and *A. pachypoda* distributed in North America and Europe often rely on beetles as effective pollinators, with their flowers frequently serving as sites for beetle mating and feeding [9,14]. During field experiments on *A. erythrocarpa*, large flies were occasionally observed visiting flowers but departed quickly. This may be related to the floral morphology of *Actaea* species, which might not adequately support pollen feeding by large flies. Therefore, the floral and inflorescence architecture of *Actaea* plants appear better suited for crawling small flies and beetles.

The hybrid mating system is also commonly present in angiosperms [16,17]. It is also common for angiosperm breeding systems to transition from cross-pollination to self-pollination [16,18]. When plants are in natural environments where cross-pollination cannot occur, self-pollination is beneficial for ensuring fertilization of ovules. Although the predominantly selfing *A. asiatica* and the predominantly outcrossing *A. erythrocarpa* are similar in floral color, staminode morphology, stigma, and anther shape, they exhibit subtle differences in sepal morphology. Compared with *A. erythrocarpa*, *A. asiatica* has a single, more tightly enclosing sepal type that does not abscise easily, whereas the sepals of *A. erythrocarpa* abscise early. Furthermore, the filaments of *A. asiatica* are coiled in the bud stage and remain curved until fully elongated after anthesis, which may be related to the persistent enclosing sepals [15]. Although both species are self-compatible, *A. asiatica* achieves selfing through the contraction of its persistent sepals, a mechanism distinct from that of *A. erythrocarpa* and other species in the *Actaea* clade [10]. In contrast, *A. erythrocarpa*, through early sepal abscission, attracts frequent pollinator visits even before anther dehiscence, thereby effectively avoiding both autogamy and geitonogamy.

Notably, *A. rubra* (i.e., *A. erythrocarpa*) exhibits geographical variation in its breeding system: populations in the United States and the Changbai Mountain population in this study both exhibit a pollinator-dependent, predominantly outcrossing system, while the Finnish population is capable of parthenogenesis [9,14]. *A. asiatica* exhibits a clearly facultative selfing system with no apomixis [10], whereas *A. spicata* and *A. pachypoda* exhibit facultative outcrossing systems [9,14]. Although pollinators provide outcrossing opportunities for the latter two species, *A. spicata* is self-incompatible and can also reproduce via parthenogenesis. Thus, the genus *Actaea* exhibits a complex and diverse array of breeding systems, encompassing outcrossing, selfing, and parthenogenesis. The expression of outcrossing and parthenogenesis in *A. erythrocarpa* across different environments can be viewed as an adaptive response.

In this study, *A. erythrocarpa* flowered relatively late in the season and had a stable pollinator assemblage, with syrphid flies and agromyzid flies identified as effective pollinators.

## 5. Conclusions

In summary, this study on the breeding system and pollination biology of *A. erythrocarpa* confirms that it is a predominantly outcrossing species reliant on insect pollination. The receptivity of the stigma occurs earlier than the detachment of the sepals. *Agromyzidae* sp. and *E. balteatus* are the main pollinators. Compared with the sympatric *A. asiatica*, differences merely in sepal morphology lead to entirely distinct breeding systems. The evolution of diverse reproductive strategies, including outcrossing, selfing, and parthenogenesis among closely related species within *Actaea* Linn. during environmental adaptation may be associated with the long-distance seed dispersal of this genus mediated by animals.

## Figures and Tables

**Table 1:** Estimation of breeding system of *A. erythrocarpa* based on OCI and P/O (means ± SE).

Items	Characteristics and Assigned Values
Diameter of a flower	10.03 ± 1.44 (8.06–12.21) mm, OCI = 3
Temporal separation of stigma receptivity and anther dehiscence	protogyny, OCI = 0
Spatial relationship of stigma and stamen	space separation, OCI = 1
OCI and breeding system	OCI = 4, self-compatible, requires pollinators
Average number of pollen in a flower	13,120.17 ± 554.14
Average number of ovule in a flower	7.39 ± 0.36
Breeding system by P/O	1773 ± 689, facultative xenogamy
Breeding system by P/O, Pellmyr, 1985 [14]	2933 ± 205, facultative xenogamy
	2057 ± 184, facultative xenogamy
	619 ± 87, facultative apomixis

**Table 2:** Percent of seed set from different treatments of *A. erythrocarpa* (means ± SE, *n* = 60, a and b represent significant differences).

Treats	Seed Set (%)
Natural pollination	85.27 ± 0.19 ^a^
Bagged after emasculation	0 ^b^
Mash bagged after emasculation	0 ^b^
Artificial self-pollination	83.78 ± 0.16 ^a^
Artificial geitonogamy after emasculation	88.54 ± 0.13 ^a^
Artificial cross-pollination after emasculation	82.65 ± 0.15 ^a^

## Data Availability

The raw data are available from the first author.
